# Analysis of Wastewater Reveals the Spread of Diverse Extended-Spectrum β-Lactamase-Producing *E. coli* Strains in uMgungundlovu District, South Africa

**DOI:** 10.3390/antibiotics10070860

**Published:** 2021-07-15

**Authors:** Siyabonga N. Gumede, Akebe L. K. Abia, Daniel G. Amoako, Sabiha Y. Essack

**Affiliations:** 1 Antimicrobial Research Unit, College of Health Sciences, University of KwaZulu-Natal, Durban 4000, South Africa; gumedes2@ukzn.ac.za (S.N.G.); essacks@ukzn.ac.za (S.Y.E.); 2 Centre for Respiratory Diseases and Meningitis, National Institute for Communicable Diseases, Johannesburg 2131, South Africa

**Keywords:** antibiotic resistance genes, antibiotic-resistant bacteria, multidrug resistance, ESBL, South Africa, wastewater treatment plants, wastewater-based epidemiology

## Abstract

Wastewater treatment plants (WWTPs) are major reservoirs of antibiotic-resistant bacteria (ARB), favouring antibiotic resistance genes (ARGs) interchange among bacteria and they can provide valuable information on ARB circulating in a community. This study characterised extended-spectrum beta-lactamase (ESBL)-producing *Escherichia coli* from the influent and effluent of four WWTPs in uMgungundlovu District, KwaZulu-Natal, South Africa. *E. coli* was enumerated using the membrane filtration method and confirmed using the API 20E test and real-time polymerase chain reaction. ESBL-producers were phenotypically identified by their susceptibility to the third-generation cephalosporins using the disc diffusion and the double-disc synergy methods against cefotaxime (30 µg) with and without 10 µg clavulanic acid. Genotypic verification was by PCR of the TEM, SHV, and CTX-M genes. The clonality of isolates was assessed by ERIC-PCR. The highest *E. coli* count ranged between 1.1 × 10^5^ (influent) and 4.3 × 10^3^ CFU/mL (effluent). Eighty pure isolates were randomly selected, ten from the influent and effluent of each of the four WWTP. ESBLs were phenotypically confirmed in 49% (*n =* 39) of the isolates, of which 77% (*n =* 30) were genotypically confirmed. Seventy-three percent of the total isolates were multidrug-resistant (MDR). Only two isolates were susceptible to all antibiotics. Overall, resistance to first and second-generation cephalosporins was higher than to third and fourth generation cephalosporins. Also, 15% of the isolates were resistant to carbapenems. The CTX-M-type ESBL (67%; *n =* 20) was the most common ESBL antibiotic resistance gene (ARG) followed by TEM (57%; *n =* 17) and SHV-types (27%; *n =* 8). Also, a substantial number of isolates simultaneously carried all three ESBL genes. ERIC-PCR revealed a high diversity of isolates. The diversity of the isolates observed in the influent samples suggest the potential circulation of different ESBL-producing strains within the studied district, requiring a more comprehensive epidemiological study to prevent the spread of ESBL-producing bacteria within impoverished communities.

## 1. Introduction

Antibiotic resistance is one of the major public health concerns globally. This is commonly demonstrated in bacteria such as *E. coli*, found on the WHO global priority list of antibiotic-resistant bacteria to guide research, discovery, and development of new antibiotics [1,2]. Antibiotic-resistant *E. coli* and antibiotic resistance genes (ARGs) have been detected in various environmental samples, including soils, surface water, and wastewater [3,4,5]. Extended-spectrum β-lactamase (ESBL)-producing bacteria, including *E. coli*, are among the most commonly encountered multidrug-resistant (MDR) bacteria today [6,7,8] and are frequently associated with a high mortality rate and prolonged hospitalisation [9,10]. This may be because clinical *E. coli* isolates frequently co-carry multiple ESBL genes with varying hydrolysis spectra to different antibiotics, leading to treatment failure [10,11,12,13].

ESBLs are a cluster of enzymes that efficiently hydrolyse third-generation oxyimino-cephalosporins such as cefotaxime, ceftazidime, and ceftriaxone and monobactams (aztreonam), but spare the cephamycins (cefotetan, cefoxitin) and carbapenems (imipenem, meropenem, ertapenem, biapenem, and doripenem) [8,14,15]. However, carbapenems, considered last-resort antibiotics used to treat severe infections caused by ESBL-producing *E. coli*, are also gradually losing their efficacy due to carbapenemases [16,17,18,19]. Most ESBL variants evolved from the parent chromosomal or plasmid-mediated Temoniera (TEM), Sulfhydryl Variable (SHV), and Cefotaximase-München (CTX-M) β-lactamases [6,7,20]. These ESBLs are among the primary causes of clinical resistance to β-lactam antibiotics in *E. coli* associated with common infections [21]. CTX-M-type ESBLs, mainly CTX-M-15, are currently the most prevalent in communities and clinical settings [7,22,23]. This family of ESBLs has superseded TEM and SHV in prevalence [6].

Wastewater treatment plants (WWTPs) have been reported as reservoirs of ESBL-producing *E. coli* and other ARB [24]. This is because of the high bacterial biomass, antibiotic metabolites, and ARGS, usually from animal and human-associated wastewater [25,26]. This cocktail of microbial species, antibiotic residues and antibiotic resistance genes (ARGs) create a favourable environment for bacterial proliferation, DNA exchange and selection of resistance among bacteria [25,27,28,29]. Given that WWTPs receive wastewater from entire communities, they can be considered as microcosms, and the analysis of wastewater from these systems could indicate the antibiotic-resistant bacteria (ARB) circulating within a given population. This has engendered the novel paradigm known as wastewater-based epidemiology as a rapid alternative public health assessment approach at the community level [30]. Data obtained from such analyses could serve as an early warning system for potential ARB-associated disease outbreaks and inform public health practitioners on the need for rapid intervention measures to present such, especially within resource-limited settings.

Thus, the current study investigated the phenotypic and genotypic characteristics of ESBL-producing *E. coli* in the influent and effluent of four WWTPs in the uMgungundlovu District, KwaZulu-Natal, South Africa. The study results would provide information regarding the diversity of ESBL-producing *E. coli* strains circulating within the community and the potential contribution of the WWTPs to these bacteria’s presence in the environment.

## 2. Results

### 2.1. Enumeration of Isolates

**Figure 1:**
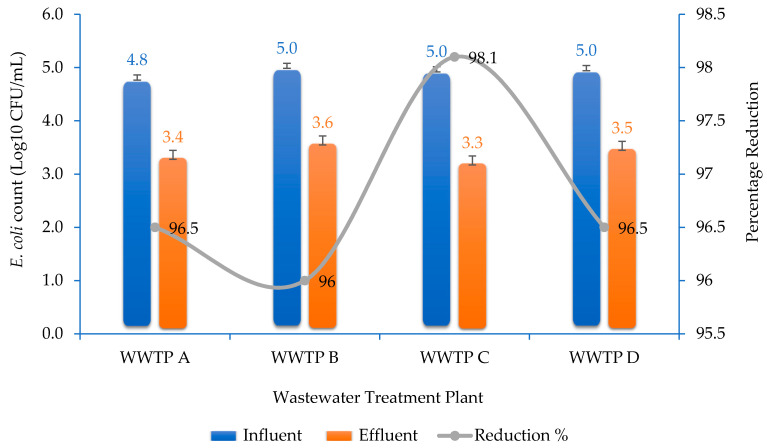
Total *E. coli* counts and percentage reduction in the studied wastewater treatment plants (WWTPs). Error bars represent the standard deviation. A–D = designation of the different WWTPs.

The total *E. coli* count in the influent water samples ranged between 6.5 × 10^4^ and 10.8 × 10^4^ CFU/mL, while in the effluent water samples, it ranged between 1.8 × 10^3^ and 4.3 × 10^3^ CFU/mL (Figure 1). Thus, the influent samples had relatively higher counts than effluent samples. In addition, all the WWTPs showed relatively high efficiency to eliminate bacterial contaminants (between 96.0–98.1%).

### 2.2. Antimicrobial Susceptibility

Resistance to the penicillins, i.e., ampicillin (94%; 75/80) and amoxicillin (88%; 70/80) was the highest except for piperacillin (57%; 46/80) and the penicillin-inhibitor combination amoxicillin-clavulanate (30%; 24/80) (Table 1). Resistance to first- and second-generation cephalosporins (cephalexin—68% [54/80]; cephalothin—66% [53/80]; cefuroxime—63% [50/80]) was higher than to third- and fourth-generation cephalosporins (ceftazidime—21% [17/80] and cefepime—20% [16]) except for cefixime (55%; 44/80) and cefotaxime (56%; 45/80). Fifteen percent of the isolates were resistant to carbapenems, while low percentage resistance was noted for nitrofurantoin (9%; 7/80), amikacin (11%; 9/80), chloramphenicol, tigecycline (16%; 13/80), and gentamicin (18%; 14/80). Variable percentage resistances were observed across the WWTPs with a general trend of higher percentages in effluent samples (Table 1).

### 2.3. Prevalence of Multidrug Resistance in E. coli Isolates

Individual isolates displayed diverse antibiograms, except for six influent isolates and thirteen effluent isolates that shared similar patterns (Table S1; Supplementary Materials). A total of 32 MDR phenotypes were evident in 73% (*n =* 58) of the isolates, with some isolates resistant to up to 20 antibiotics (Table S1; Supplementary Materials). However, effluent isolates (93%; *n* = 37) had a relatively higher number of MDR isolates than influent isolates (53%; *n* = 21). Most MDR isolates (76%) demonstrated resistance to at least 50% (11 or more) of the tested antibiotics. Of these, nine isolates were extremely drug-resistant, with resistance to more than twenty antibiotics. The remainder (24%) showed resistance profiles ranging from three up to ten antibiotics simultaneously. Twenty-five percent of the isolates were resistant to at least two antibiotics or more but were not assigned as MDR, and only two isolates were susceptible to all antibiotics.

### 2.4. ESBL Resistance Genes

Of the 39 (49%) ESBL-positive *E. coli* isolates, ESBL ARGs were detected in 77% (*n* = 30), with more being isolated from effluent samples (*n* = 19) compared to influent samples (*n* = 11). The CTX-M-type ESBL (67%; *n* = 20) was the most prevalent ARG followed by TEM (57%; *n* = 17) and SHV-type (27%; *n =* 8). Ten percent (*n* = 3) of isolates co-carried CTX-M and TEM-type ARGs, 13% (*n* = 4) co-carried CTX-M and SHV ARGs, and 13% (*n* = 4) carried all the three ESBL types. Nine isolates (30%) carried CTX-M alone, 33% (*n* = 10) carried TEM alone, and no isolates carried SHV alone (Table 2).

### 2.5. Strain Typing by ERIC-PCR

**Figure 2:**
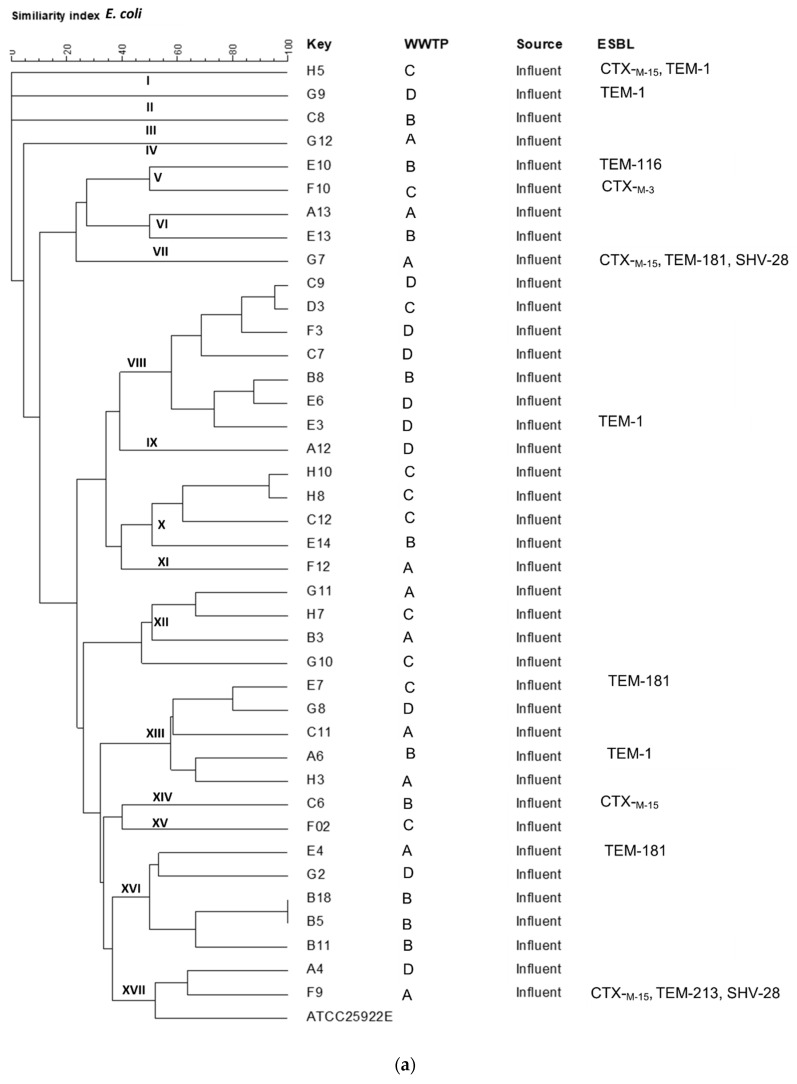

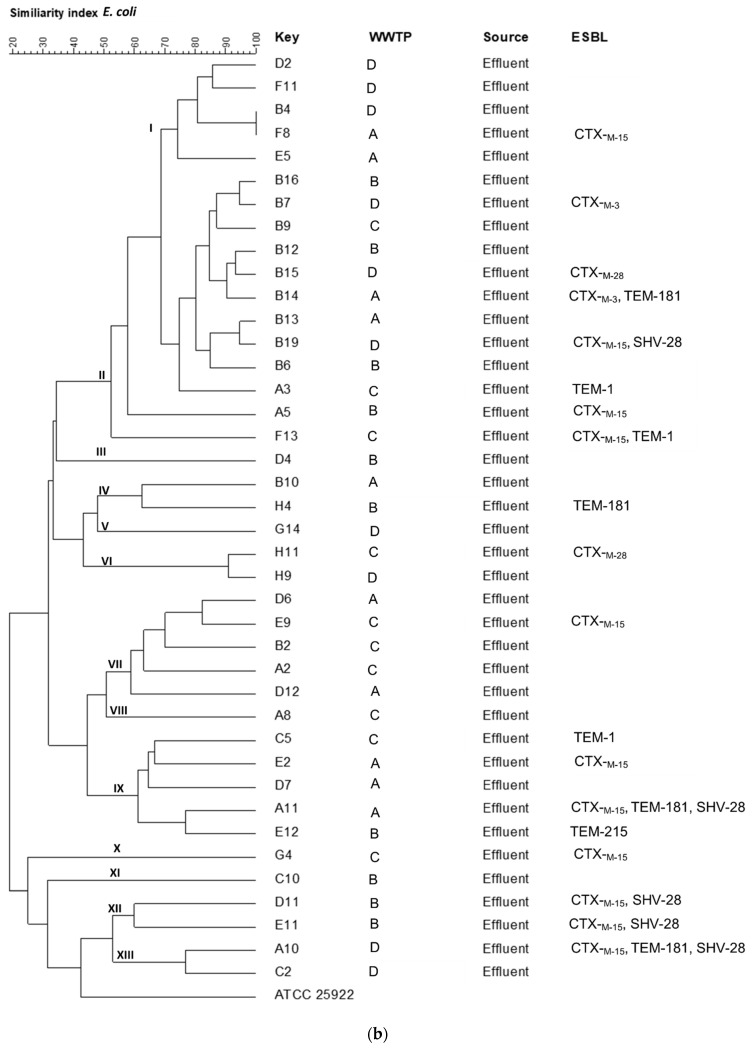
Dendrogram of ESBL-producing *E. coli* from the (**a**) influent and (**b**) effluent of the wastewater treatment plants (WWTPs). I–XVII = ERIC Types. A–D = Designation of the different WWTPs.

The Enterobacterial Repetitive Intergenic Consensus (ERIC)-PCR analysis of the *E. coli* isolated from influent (*n* = 40) and effluent (*n* = 40) water samples showed high diversity (Figure 2a and Figure 2b, respectively). A similarity index of ≥50% was used to group the isolates into different ERIC-types. Influent *E. coli* isolates were classified into 17 ERIC-types (Ⅰ-XVII), while effluent *E. coli* strains were classified into 13 ERIC-types (Ⅰ-XIII). From the influent *E. coli* isolates three major ERIC-types were identified, viz VIII (*n* = 7), XIII (*n* = 5) and XVI (*n* = 5) while in the effluent *E. coli* isolates four major ERIC-types were identified, viz I (*n* = 5), II (*n* = 12), VII (*n = 5*), IX (*n* = 5). Major ERIC-types from influent *E. coli* consisted of isolates from different WWTPs, with A, B, and D isolates falling under the same ERIC-types. Major ERIC-types from effluent *E. coli* isolates consisted of a mixture of isolates from all sampling sites.

## 3. Discussion

The analysis of wastewater can indicate the resistant bacterial species circulating within a community. Also, the release of ARGs and ARB into aquatic environments may create environmental reservoirs of ARB and ARGs, posing significant challenges to human health [31] and the evolution of the environmental microbial population [25,32,33]. This study demonstrated diverse ESBL-producing *E. coli* circulating within South African communities and the subsequent discharge of these bacteria by WWTPs into receiving surface waters.

### 3.1. Enumeration of E. coli

The average total *E. coli* count in the influent water samples was about 10^5^ CFU/mL in all WWTPs, and the effluent counts were smaller by up to two orders of magnitude than raw influent. The low *E. coli* count in the effluent indicates the WWTPs’ process efficiency, with bacterial reduction of more than 96% across all WWTPs (Table 1). However, significant bacterial biomass, including some ARB, was released with the effluent into surrounding surface water bodies. Nevertheless, the highest microbial load in all the four WWTPs’ effluents were below the limits of 10^5^ CFU/100 mL of effluent wastewater stipulated in the South African National Water Act [34].

### 3.2. Antibiotic Susceptibility

Antibiotic sensitivity testing of *E. coli* isolates showed variable percentages of resistance across the WWTPs with a general trend of higher percentages in effluent samples (Table 1). Ampicillin is highly used in human and veterinary medicine for treatments and prophylaxis [35]. Its ready availability and extensive use could have favoured the development of resistance to this antibiotic. Interestingly, previous studies within the same locality have reported high resistance to ampicillin in humans [36], animals (poultry and pigs) [37,38], and the environment (river water) [39]. Together with the current study’s findings, these reports are substantial evidence that resistance to penicillins is of public health significance within the uMgungundlovu District. Disinfection by chlorination used in WWTPs alters the microbial community composition [40,41] but may also contribute to selecting antibiotic-resistant *E. coli* [42]. Chlorine tolerance and chlorine-induced upregulation of ARGs in bacteria have been associated with resistance to β-lactams and other antibiotics [42]. Resistance to ampicillin (94%) and amoxicillin (88%) was the highest in this study, with increased percentages evident in chlorine-treated effluent samples in all WWTPs. This finding is further supported by the fact that a high percentage of resistance to these antibiotics had previously been reported in one of the main rivers that serve as a receiving water body for some of the WWTPs studied [39].

### 3.3. Multidrug Resistance and ESBL Production

ESBL-production is among the commonly encountered mechanisms for MDR in bacteria [6,7]. The phenotypically determined presence of ESBL in 49% of isolates was genotypically corroborated in 31% of the isolates, and five isolates carried TEM-1 ARG (Table 2). The variation could be attributed to unexplored ESBLs or other resistance mechanisms, such as the possible presence of unexamined AmpC β-lactamase in some of the isolates. Six isolates that were genotypically negative for ESBL-production showed resistance to cefoxitin associated with AmpC cephalosporinase activity. Overexpressed AmpC β-lactamase can either mask the phenotypic detection of an ESBL or present as an ESBL-phenotype in the absence of an ESBL [43]. Moreover, other studies have also indicated the discrepancy of phenotypic methods of ESBL detection [44,45,46]. For example, Ojer-Usoz et al. [45] examined WWTP water samples in Navarra, Spain. They identified ESBL-producing *Enterobacterales* (*n* = 185) using Chrom ID ESBL agar plates (100%), with varying levels of detection compared to the combination disk test (86.5%), double-disk synergy test (75.7%), and PCR (71.3%) as a confirmatory method. As a result, molecular techniques remain the gold standard in the identification of ESBL-producing bacteria.

The prevalence of ESBL-producing *E. coli* in this study was similar to the findings of Nzima et al. [24], who reported an ESBL prevalence of 28.6% in *E. coli* isolated from WWTP and effluent-receiving river in Durban, South Africa. The β-lactamase-producers were relatively higher in the effluent isolates (48%; *n* = 19) than influent isolates (28%; *n* = 11). A recent multi-country study (Denmark, Spain, and the United Kingdom) revealed that although wastewater arising from urban areas may contain large amounts of ESBL and carbapenamase-associated ARGs, their concentrations were drastically reduced within the sewer systems, resulting in lower values during sewer convergence [47]. This observation could explain the lower ESBL prevalence in the influent samples in our study. However, unlike [47] that further reported that the ESBL ARG concentration dropped even further after the treatment process, the current study results revealed that the WWTP favoured the proliferation of ESBL-producing bacteria, resulting in higher counts in the effluent samples. These discrepancies could be due to the sample size and the sampling regime used, as the current study was a point-prevalence study within a single locality, compared to the multi-country approach used in the previous study. Our findings were nevertheless similar to a previous study [48], that reported a higher prevalence of ESBL-producing *E. coli* in the effluent samples (0.6%) compared to the influent samples (0.3%) recovered in a WWTP in Besançon, France, indicating a higher tolerance of ESBL-producers to the water treatment protocol of the WWTP for the elimination of bacteria. The increased frequency of ESBL-producers in the effluent could also be attributed to high bacterial biomass and nutrient levels in the WWTPs, favouring proliferation and interchange of ARGs among bacteria [49,50].

ESBL-producing *E. coli* isolates demonstrated varying resistance percentages to cephalosporins irrespective of the ESBL-type or variant they carried. ESBL-positive isolates were resistant to at least one or more cephalosporins tested, with higher resistance observed in the first and second-generation cephalosporins (>76%). The highest resistance to the third- and fourth-generation cephalosporins was observed to cefixime (72%) and cefotaxime (80%) compared to ceftazidime and cefepime (52%). Isolates carrying a gene that encodes for CTX-M-28 and CTX-M-3 variants demonstrated a similar hydrolytic efficiency for cephalosporins (i.e., LEX-CEF-CFM-CXM-CTX) except for one CTX-M-3-producing isolate, which was resistant to all cephalosporins. Ten isolates that carried one ARG or co-carried multiple ARGs viz *bla*_CTX-M-15_, *bla*_SHV-28_, *bla*_TEM-181_ (*n =* 3), *bla*_CTX-M-15_, *bla*_SHV-28_, *bla*_TEM-213_ (*n =* 1), *bla*_CTX-M-15_, *bla*_SHV-28_ (*n = 4*), *bla*_TEM-116_ (*n =* 1) and *bla*_CTX-M-15_ (*n =* 1) also demonstrated resistance to all the cephalosporins tested. The World Health Organization listed late-generation cephalosporins among priority antibiotics for human medicine [51], and thus the occurrence and spread of bacterial strains resistant to these and other antibiotics present a serious clinical threat. ESBL-producing *E. coli* were isolated in influent samples leading from WWTPs A and D, which process hospital wastewater. Thus, the isolates may be of hospital origin, further highlighting the role of WWTPs as a proxy for the types of ESBL-producing strains circulating within the community. This necessitates the pre-treatment of hospital effluent using methods such as coagulation-flocculation and flotation [52] before discharge into the municipal sewage system [26].

WWTPs receive animal and human-associated wastewater frequently containing contaminants such as metals, biocides, disinfectants, antibiotics, ARGs, and high bacterial biomass [25,29,33,53]. The pressure by these contaminants on microbial populations in the WWTPs may exert selection pressure that can promote upregulation or enrichment of ESBL and other ARGs carried by bacteria through selection or co-selection. Studies have indicated that ESBL-producers demonstrate co-resistance to other antibiotic classes, including fluoroquinolones, aminoglycosides, tetracyclines, and trimethoprim/sulfamethoxazole [32,45]. Resistance to ciprofloxacin (60%), nalidixic (72%), tetracycline (88%), and trimethoprim/sulfamethoxazole (92%) was observed among ESBL-producing isolates in the current study. Similarly, Dolejska et al. [54] reported high resistance to ciprofloxacin (100%), nalidixic (100%), tetracyclines (73%), and trimethoprim/sulfamethoxazole (73%) in ESBL-positive *E. coli* isolated from a WWTP in the Czech Republic. Nzima et al. [24] also reported high resistance to tetracyclines (80%) in ESBL-producing *E. coli* isolates recovered in a WWTP in Durban, South Africa. Isolates that produced CTX-M-3 and CTX-M-28 were resistant to fluoroquinolones, and CTX-M-15-producing isolates demonstrated varying percentages of resistance to this antibiotic class. Non-ESBL TEM-1-producing isolates also demonstrated some resistance to these antibiotics. Co-resistance to aminoglycosides (36%) was also observed in some ESBL-producing *E. coli,* but resistance to this antibiotic class was generally low. Lower resistance percentages were noted for nitrofurantoin, chloramphenicol, tigecycline, and carbapenems (Table S1; Supplementary Materials).

The CTX-M-type ESBLs were the most prevalent ARGs detected in this study (Table 2). CTX-Ms are currently the most prevalent ESBLs, classified into six subgroups [55,56]. The predominance of the CTX-M ESBL observed in *E. coli* isolates in the current study was similar to a previous study [32] that found a higher prevalence of CTX-M producing *E. coli* isolates in the effluent samples (100%) of a WWTP in Curitiba, Brazil. This indicates that WWTPs are a microcosm of ARGs circulating within a community and a hotspot for proliferation and dissemination of CTX-M-producing *E. coli* in the environment via effluent discharge.

CTX-M-type ESBLs belonging to the CTX-M-1 sub-lineage were detected in this study. The sub-lineages CTX-M-1 and CTX-M-9 constitute the most common variants within the CTX-M family [55,57]. CTX-M-15, the most globally dispersed genotype within the CTX-M family [8,58], was the most frequently detected ESBL variant mainly detected in effluent water samples in all WWTPs. CTX-M-15-producing bacteria are clinically relevant [59], and the presence of CTX-M-15 producing *E. coli* in WWTP effluent implicates isolates of clinical origin, hence a picture of their presence in the study community. Furthermore, some CTX-M-15 producing *E. coli* isolates co-carried other ESBLs genes, a common phenomenon among clinical isolates that may increase drug resistance. Studies have indicated that the global prevalence of CTX-M-15 could be attributed to specific clones [60]. However, the CTX-M-15 producing *E. coli* detected in this study were evident in multiple isolates.

### 3.4. Clonality of Isolates

ERIC-PCR fingerprinting of *E. coli* isolates using a similarity index of ≥50% showed a high diversity of *E. coli* strains. A higher number of clonal clusters was observed in the influent sample than the effluent sample (Figure 1). This could be attributed to the fact that the sewage network leading to WWTPs is vast, consisting of diverse bacterial strains of different origins, suggesting that different ESBL-producing *E. coli* strains could be circulating within the different communities they serve. This was further supported by the decline in the number of clonal clusters in the effluent isolates. The WWTPs used the same treatment processes resulting in the observed decrease in clonal clusters. The decreased clonal clusters in the effluent samples could also be attributed to some clones being more susceptible to the wastewater treatment processes of the WWTP for the elimination of bacteria. Despite the high diversity, some *E. coli* isolates recovered from influent samples of WWTP A, B and D showed some similarity in major ERIC-types XVI and XIII. Similar ERIC-types in geographically distinct collection points indicate the endemicity of certain clones in the uMgungundlovu District communities. More than 60% of the influent *E. coli* isolates from the same sampling point of the three WWTPs (A, B, and C) fell under different ERIC-types, except for the clonal cluster designated as ERIC-type VIII, which constituted of several isolates from WWTP D influent sample, indicating the predominance of this clone in the region around this WWTP.

While the current study reveals that diverse ESBL-producing *E. coli* clones circulate within the studied community, the presence of these bacteria was not determined in the surrounding aquatic environment. Further studies that would also sample the receiving water bodies into which these WWTPs discharge their effluent could provide more information on whether these resistant bacteria are present in rivers within the community. Ascertaining this would help prevent the risk of infection with resistant bacteria in parts of the community using the water directly for different needs.

## 4. Materials and Methods

### 4.1. Study Sites

The study site consisted of four wastewater treatment plants (A–D) in the uMgungundlovu District. WWTPs A and D treat industrial wastewater, hospital wastewater, and domestic wastewater from 117,303 of the 679,039 persons with flush toilets connected to the sewage system within Msunduzi local municipality. These WWTPs have an average inflow capacity of 76 (A) and 0.5 (D) megalitres/day. WWTP B has an inflow capacity of 5.6 megalitres/day, including industrial wastewater, hospital wastewater, and domestic wastewater from 31,322 of the 109,867 persons with flush toilets connected to the sewage system within uMngeni local municipality. WWTP C has an inflow capacity of 0.54 megalitres/day, mainly domestic wastewater from 11596 persons residing in the surrounding suburbs. These WWTPs use aeration basins for biological nutrient removal, clarifiers for the separation process, and chlorine disinfection.

### 4.2. Sampling

Water samples were collected once-off from the WWTPs’ influent in the primary sedimentation tank, while the effluent samples were collected at the treated wastewater discharge point in the chlorine contact tank. Samples were collected using sodium thiosulfate (20 mg/L) coated sterile 500 mL bottles (JVL Laboratories, Durban, South Africa), following standard procedures prescribed for examining wastewater [61]. Water samples were transported to the Antimicrobial Research Unit (ARU), University of KwaZulu-Natal, in a portable 4 °C cooler-box for processing within six hours from collection.

### 4.3. Quantitative Analysis and Isolate Identification

Ten millilitres (10 mL) of each water sample were serially diluted (10^1^ to 10^6^) in 90 mL of sterile distilled water. A thoroughly homogenised mixture was vacuum filtered through a 0.45 µm pore membrane filter (Pall Corp, Port Washington, NY, USA) using the Sterifil Aseptic System apparatus (Pall Corp). The membrane filter was aseptically inoculated onto eosin methylene blue agar plates (Oxoid™, Waltham, MA, USA) and incubated at 37 °C for 24 h. Colonies with morphology and colour characteristics of *E. coli* (i.e., green metallic sheen with dark centres) were counted in each plate and recorded as colony forming units per millilitre (CFU/mL). Randomly selected presumptive *E. coli* colonies were sub-cultured on nutrient agar (Oxoid™) and incubated at 37 °C for 18–24 h, after which isolates were phenotypically identified using the API 20E test kits (BioMerieux, Marcy-l’Étoile, France). Ten putative *E. coli* isolates per sampling point were randomly selected and individually stored in a Muller Hinton broth: glycerol (70:30) mixture in cryovials at −80 °C for further processing, giving 40 influent and 40 effluent isolates from the four WWTPs.

### 4.4. Molecular Confirmation of E. coli by Real-Time Polymerase Chain Reaction (PCR)

Genomic DNA of overnight Muller Hinton broth (Oxoid™) cultures (20 h at 37 °C) was extracted using the GeneJet Genomic DNA purification kit (ThermoFisher Scientific™, Waltham, MA, USA) to detect the presence of the *E. coli* housekeeping gene, malate dehydrogenase (*mdh*), using real-time PCR. DNA purity and concentration were determined using a NanoDrop^TM^ 8000 spectrophotometer (Thermo Scientific, Wilmington, DE, USA) at 260/280 nm wavelength. DNA samples were subsequently stored at −20 °C for further examination.

PCR assays were performed in a 10-µL final volume comprising of 5 µL of Luna^®^ Universal qPCR Master Mix (New England BioLabs^®^ Inc., Ipswich, MA, USA), 1 µL of nuclease-free water (ThermoFisher Scientific™), 0.5 µL of each forward and reverse primer at a final concentration of 0.5 µM (Inqaba Biotechnical Industries (Pty) Ltd., Pretoria, South Africa) and 3 µL of template DNA. The primers used were *mdh*-F (5′GGTATGGATCGTTCCGACCT-3′) and *mdh*-R-(5′-GGCAGAATGGTAACACCAGAGT-3′) [62], under conditions previously described [63]. All reactions were run in a QuantStudio^TM^ 5 Applied Biosystems real-time thermocycler (ThermoFisher Scientific™). *E. coli* ATCC 25922 (American Type Culture Collection, ATCC, Manassas, VA, USA) was used as a positive control. The no template control contained all the reagents except template DNA. Results were interpreted by melt curve analysis using the QuantStudio Design and Analysis software version 1.4.3 (ThermoFisher Scientific™) under default mode.

### 4.5. Antibiotic Susceptibility Testing and Determination of ESBL Production

Antibiotic sensitivity profiles of *E. coli* isolates were examined against 23 antibiotics using the standard disc diffusion method [64]. The following antibiotic panel (Oxoid™) was used: amoxicillin/clavulanic acid (AMC 20/10 μg), amoxicillin (AMX 10 μg), amikacin (AMK 30 μg), ampicillin (AMP 10 μg), cefepime (FEP 30 μg), cephalothin (CEF 30 μg), cefotaxime (CTX 30 μg), cefoxitin (FOX 30 μg), cefixime (CFM 5 μg), ceftazidime (CAZ 30 μg), cephalexin (LEX 30 μg), cefuroxime (CXM 30 μg), chloramphenicol (CHL 30 μg), ciprofloxacin (CIP 5 μg), gentamicin (GEN 10 μg), imipenem (IPM 10 μg), meropenem (MEM 10 μg), nitrofurantoin (NIT 100 μg), piperacillin (PIP 100 μg), tetracycline (TET 30 μg), tigecycline (TGC 15 μg), nalidixic (NAL 30 μg) and trimethoprim/sulfamethoxazole (SXT 1.25/23.75 μg). Susceptibility profiles were determined according to the Clinical Laboratory Standard Institution (CLSI) guidelines [65], and *E. coli* ATCC 25922 was used for quality control. The double-disc synergy test [66] using 30 µg cefotaxime in the presence and absence of 10 µg clavulanic acid (MAST Group Ltd., Bootle, UK) was undertaken to identify ESBL-producing isolates. *E. coli* isolates displaying resistance to one or more antibiotics from three different antibiotic classes were assigned as multidrug-resistant (MDR) isolates.

### 4.6. Genotypic Characterisation of ESBLs

The previously extracted template DNA was used to detect ESBLs-encoding genes (*bla*_CTX-M_ and *bla*_SHV_) by real-time PCR. These reactions were carried out in a 25 µL final volume consisting of 12.5 µL of Luna^®^ Universal qPCR Master Mix (New England BioLabs^®^ Inc., Ipswich, MA, USA), 5.5 µL of nuclease-free water (ThermoFisher Scientific™), 1 µL of each forward and reverse primer with a final concentration of 1 µM and 5 µL of template DNA. Amplification reactions were conducted in a QuantStudio^TM^ 5 Applied Biosystems (ThermoFisher Scientific™) using specific primer sets (Inqaba Biotechnology Industries (Pty) Ltd.) (Table S2; Supplementary Materials). The cycling conditions included a hot-start initial activation at 98 °C for 50 s, followed by 35 cycles of denaturation at 95 °C for 10 s, final extension at 72 °C for 20 s and annealing temperature at 58 °C for 1 min. Results were interpreted by melt curve analysis using the QuantStudio Design and Analysis software version 1.4.3 (ThermoFisher Scientific™).

The TEM gene was amplified in a Thermal Cycler T100^TM^ (Bio-Rad, Hercules, CA, USA) in 25 µL final volume using 12.5 µL of DreamTaq Green PCR Master Mix (ThermoFisher Scientific™), 5.5 µL of nuclease-free water (ThermoFisher, Scientific™), 1 µL of each forward and reverse primer (Inqaba Biotechnology Industries (Pty) Ltd.) at a final concentration of 1 µM and 5 µL of template DNA. PCR conditions were an initial activation at 94 °C for 3 min, and 35 cycles of denaturation at 94 °C for 1 min, annealing at 55 °C for 1 min, extension at 72 °C for 90 s and a final extension at 72 °C for 7 min. Five microliters of each PCR product was subsequently electrophoresed on a 1.5% agarose gel for 3 h at 70 V using 1 × Tris-acetate EDTA (TAE) buffer and stained for 30 min by immersing in 0.1 mg/mL ethidium bromide solution. Amplicons were visualised using a Bio-Rad molecular imager Gel Doc^TM^ XR+ imaging system (Bio-Rad). Previously confirmed *bla*_CTX-M_, *bla*_TEM,_ and *bla*_SHV_ positive isolates from the ARU laboratory collections were used as internal positive controls. The no template control, consisting of all reagents except DNA, was concurrently run during amplification reactions. PCR products (20 µL) of CTX-M-positive isolates (*n =* 20), TEM-positive isolates (*n =* 17), and SHV-positive isolates (*n =* 8) were sequenced using the Sanger sequencing method at Inqaba Biotechnology Industries (Pty) Ltd. to identify ESBL genes and ascertain point mutations conferring novelty (if any). Sequence results were interpreted using Chromas (http://technelysium.com.au/wp/chromas/, accessed on 26 April 2020) and BioEdit (https://bioedit.software.informer.com, accessed on 26 April 2020) software.

### 4.7. Strain Typing by ERIC-PCR

The Enterobacterial Repetitive Intergenic Consensus (ERIC)-PCR assay was executed in a 20 µL final volume comprising of 16 µL of DreamTaq PCR Master Mix (2X) (ThermoFisher Scientific™), 1 µL of nuclease-free water, 0.5 µL of Eric 1 primer (ATGTAAG CTCCTGGGGATTCAC) and 0.5 µL of Eric 2 primer (AAGTAAGTGACTGGGGT GAGCG) (Inqaba Biotechnology Industries, (Pty) Ltd.) [67] and 3 µL of template DNA. Amplification reactions were conducted in a Bio-Rad T100^TM^ thermal cycler (Bio-Rad) under the following cycling conditions: initial activation at 94 °C for 3 min, 30 cycles of denaturation at 94 °C for 30 s, annealing at 50 °C for 1 min, extension at 65 °C for 8 min and a final extension at 65 °C for 16 min. Five microliters of each PCR product was subsequently electrophoresed on a 1% agarose gel for 3 h at 70 V using 1 × Tris-acetate EDTA (TAE) buffer and stained for 30 min by immersing in 0.1 mg/mL ethidium bromide solution. Amplicons were visualised using the Bio-Rad molecular imager Gel Doc^TM^ XR+ imaging system (Bio-Rad), and *E. coli* ATCC 25922 was used as a positive control. Captured gel images were analysed using the BioNumerics software version 6.6 (Applied Maths NV, Sint-Martens-Latem, Belgium) as previously described by McIver et al. [37]. Clusters were determined at a 50% similarity cut-off value [68].

## 5. Conclusions

The current study demonstrated the ubiquity of antibiotic-resistant ESBLs-producing *E. coli* in the uMgungundlovu District by examining four major WWTPs. Despite the high removal efficiency observed in the WWTPs, substantial ARB numbers were discharged into the receiving water bodies through the effluents, which may present a route for wide dispersion of ESBLs-producing *E coli*. This poses a public health threat to communities who use waters contaminated with WWTPs effluent discharge for their domestic water demands. The high prevalence of clinically relevant CTX-M-15 producing *E. coli* in WWTPs indicates they might be of clinical origin. Therefore, while measures are developed and implemented to curb the problem of AMR, specifically those linked to ESBL, within communities in South Africa, the need for pre-treatment of hospital wastewater before it is channelled to municipal WWTPs should be highlighted to minimise transmission.

## Figures and Tables

**Table 1:** Overall percentage resistance of *E. coli* to different antibiotics.

Antibiotics	A		B		C		D	
	Influent	Effluent	Influent	Effluent	Influent	Effluent	Influent	Effluent
FEP	30	30	30	20	10	10	10	20
CAZ	30	30	30	30	10	10	10	20
CTX	30	50	40	90	20	70	60	90
CFM	40	50	40	90	20	60	50	90
FOX	50	20	30	20	10	10	10	20
LEX	50	60	80	80	30	60	80	100
CXM	30	60	50	100	20	90	60	90
CEF	30	60	60	100	30	80	70	100
MEM	30	10	20	20	10	10	0	20
IPM	30	10	20	20	10	10	0	20
AMK	20	10	20	20	10	10	0	20
GEN	20	20	20	20	10	20	10	20
TGC	30	20	20	20	10	10	0	20
TET	30	80	60	100	20	100	80	90
NAL	40	80	50	90	30	70	70	90
CIP	20	50	30	90	20	60	70	80
CHL	50	20	20	0	10	20	0	10
NIT	20	10	0	10	10	10	0	10
SXT	70	90	60	100	40	90	90	90
AMC	50	20	30	20	20	20	10	30
AMX	70	90	80	100	80	100	90	90
PIP	30	70	30	80	40	60	60	80
AMP	90	90	90	100	80	100	100	100

A–D = wastewater treatment plants; AMC = amoxicillin/clavulanic acid, AMX = amoxicillin, AMK = amikacin, AMP = ampicillin, FEP = cefepime, CEF = cephalothin, CTX = cefotaxime, FOX = cefoxitin, CFM = cefixime, CAZ = ceftazidime, LEX = cephalexin, CXM = cefuroxime, CHL = chloramphenicol, CIP = ciprofloxacin, GEN = gentamicin, IPM = imipenem, MEM = meropenem, NIT = nitrofurantoin, PIP = piperacillin, TET = tetracycline, TGC = tigecycline, NAL = nalidixic acid, SXT = trimethoprim/sulfamethoxazole.

**Table 2:** Distribution of the β-lactamase variants among the isolates from the different WWTPs.

		β-Lactamase Enzymes Detected		
WWTP	Sampling Point	CTX-M-Type Variants (*n*)	SHV-Type Variants (*n*)	TEM-Type Variants (*n*)
A	Influent	CTX-M-15 (2)	SHV-28 (2)	TEM-181 (2) TEM-213 (1)
	Effluent	CTX-M-15 (3) CTX-M-3 (1)	SHV-28 (1)	TEM-181 (2)
B	Influent	CTX-M-15 (1)	Not detected	TEM-116 (1) TEM-1 (1)
	Effluent	CTX-M-15 (3)	SHV-28 (2)	TEM-181 (1) TEM-215 (1)
C	Influent	CTX-M-15(1) CTX-M-3 (1)	Not detected	TEM-1 (1) TEM-181 (1)
	Effluent	CTX-M-28 (1) CTX-M-15 (3)	SHV-28 (1)	TEM-1 (3)
D	Influent	Not detected	Not detected	TEM-1 (2)
	Effluent	CTX-M-3 (1) CTX-M-28 (1) CTX-M-15 (2)	SHV-28 (2)	TEM-181 (1)
TOTAL		**20**	**8**	**17**

## Data Availability

All data related to this work have been included in the manuscript and its accompanying supplementary materials.

## Supplementary Materials

The following are available online at https://www.mdpi.com/article/10.3390/antibiotics10070860/s1, Table S1: Multidrug-resistant *E. coli* phenotypes identified in the study, Table S2: Primer sets for the amplification of TEM, SHV, and CTX-M-type ESBLs.
