# Supplementary material for: Analysis of Wastewater Reveals the Spread of Diverse Extended-Spectrum β-Lactamase-Producing E. coli Strains in uMgungundlovu District, South Africa
Source: Antibiotics (Basel). 2021 Jul 15;10(7):860. doi: 10.3390/antibiotics10070860 (PMC8300763; doi:10.3390/antibiotics10070860)
Supplement: Supplementary file 1 [file antibiotics-10-00860-s001.zip › antibiotics-1282687-supplementary.pdf]

Supplementary Materials

Analysis of Wastewater Reveals the Spread of Diverse Extended-spectrum β-Lactamase-producing *E. coli* Strains in uMgungundlovu District, South Africa

Siyabonga N. Gumede [1], Akebe L. K. Abia [1,*], Daniel G. Amoako [1,2*], Sabiha Y. Essack[1]

[1] Antimicrobial Research Unit, College of Health Sciences, University of KwaZulu-Natal, Durban 4000, South Africa; (S.N.G.); lutherkinga@yahoo.fr (A.L.K.A.); amoakodg@gmail.com (D.G.A.); essacks@ukzn.ac.za (S.Y.E.)

[2] Centre for Respiratory Diseases and Meningitis, National Institute for Communicable Diseases, Johannesburg 2131, South Africa

* Correspondence: lutherkinga@yahoo.fr (A.L.K.A.); amoakodg@gmail.com (D.G.A.)

Table S1. Multidrug-resistant E. coli phenotypes identified in the study.

MDR PHENOTYPES OBSERVED IN ISOLATES	WATEWATER TREATMENT PLANTS							
	WWTP A		WWTP B		WWTP C		WWTP D	
	Inf	Eff	Inf	Eff	Inf	Eff	Inf	Eff
**FOX-LEX-CHL-NAL-SXT**	1							
**PIP-TET-AMX-NAL-AMP-SXT**		1						
**CHL-TET-CEF-CXM-AMX-AMP-SXT**		1						
**PIP-TET-CEF-CXM-AMX-AMP-SXT-CTX**						1		
**TET-CFM-CXM-AMX-CIP-NAL-AMP-SXT-CTX**						1		
**LEX-TET-AMX-AMP-SXT**			1					
**TET-CEF-CXM-AMX-AMP-SXT**						1		
**LEX-CHL-TET-CXM-AMX-CIP-NAL-AMP**						1		
**FOX-LEX-CAZ-FEP-CEF-CFM-CXM-AMX-NAL-AMP**			1					
**FOX-AMC-AMK-LEX-CHL-GEN-MEM-CAZ-FEP-TGC-PIP-TET-CEF-CFM-CXM-AMX-CIP-NAL-AMP-SXT-IMP-CTX**			1					
**LEX-TET-AMX-CIP-NAL-AMP**							1	
**AMC-CHL-PIP-TET-CEF-AMX-AMP-SXT**						1		
**FOX-AMC-AMK-LEX-GEN-MEM-CAZ-FEP-TGC-NIT-PIP-TET-CEF-CFM-CXM-AMX-NAL-AMP-SXT-IMP-CTX**						1		
**PIP-TET-CEF-CFM-CXM-AMX-CIP-NAL-AMP-SXT-CTX**				1				
**TET-CEF-AMX-AMP-SXT**							1	
**TET-CEF-CXM-AMX-AMP-SXT**			1					
**CAZ-PIP-TET-CEF-CXM-AMX-CIP-NAL-AMP-SXT**				1				
**LEX-TET-CEF-CFM-CXM-AMX-CIP-NAL-AMP-SXT-CTX**				2				1
**LEX-GEN-PIP-TET-CEF-CFM-CXM-AMX-CIP-NAL-AMP-SXT-CTX**						1		
**AMC-LEX-CHL-PIP-TET-CEF-CFM-CXM-AMX-CIP-NAL-AMP-SXT-CTX**								1
**LEX-CHL-TGC-PIP-TET-AMX-NAL-AMP-SXT**		1						
**FOX-AMC-AMK-LEX-GEN-MEM-CAZ-FEP-TGC-**				1				1

**PIP-TET-CEF-CFM-CXM-AMX-AMP-SXT-IPM-CTX**								
**LEX-TET-CEF-CFM-CXM-AMX-CIP-NAL-AMP-SXT-CTX**						1		
**FOX-AMC-LEX-CHL-MEM-CAZ-FEP-TGC-PIP-TET-CEF-CFM-CXM-AMX-AMP-SXT-IMP-CTX**	1							
**LEX-PIP-TET-CEF-CFM-CXM-AMX-CIP-NAL-AMP-SXT**					1			
**FOX-AMC-AMK-LEX-CHL-GEN-MEM-CAZ-FEP-TGC-NIT-PIP-TET-CEF-CFM-CXM-AMX-CIP-NAL-AMP-SXT-IMP-CTX**	2							
**FOX-AMC-LEX-CFM-AMX-NAL-AMP-SXT**	1							
**FOX-AMC-AMK-LEX-GEN-MEM-CAZ-FEP-TGC-NIT-PIP-TET-CEF-CFM-CXM-AMX-CIP-NAL-AMP-SXT-IMP-CTX**		1		1	1			1
**LEX-PIP-TET-CEF-CFM-CXM-AMX-CIP-NAL-AMP-SXT-CTX**		2	1	4		2	5	5
**FOX-AMC-AMK-LEX-GEN-MEM-CAZ-FEP-TGC-PIP-TET-CEF-CFM-CXM-AMX-CIP-NAL-AMP-SXT-IMP-CTX**			1					
**LEX-CAZ-FEP-PIP-TET-CEF-CFM-CXM-AMX-CIP-NAL-AMP-SXT-CTX**		1						
**FOX-AMC-LEX-GEN-CAZ-FEP-PIP-TET-CEF-CFM-CXM-AMX-CIP-NAL-AMP-SXT-CTX**		1					1	
**TOTAL**	**5**	**8**	**6**	**10**	**2**	**10**	**8**	**9**

AMC = Amoxicillin/clavulanic acid, AMX = amoxicillin, AMP = ampicillin, PIP = piperacillin, CEF = cephalothin, LEX = cephalexin, FOX = cefoxitin, CXM = cefuroxime, CFM = cefixime, CTX = cefotaxime, CAZ = ceftazidime, FEP = cefepime, IMP = imipenem, MEM = meropenem, TET = tetracycline, TGC = tigecycline, AMK = amikacin, GEN = gentamicin, CIP = ciprofloxacin, NAL = nalidixic acid, CHL = chloramphenicol, NIT = nitrofurantoin, SXT = trimethoprim/sulfamethoxazole, Inf = Influent, Eff = Effluent.

Table S2: Primer sets used for the amplification of TEM, SHV, and CTX-M-type ESBLs.

Target enzyme	Primer	Sequence 5′ to 3′	Amplicon size (bp)	Authors (Year) [Reference]
TEM	TEMMF	TTACCAATGCTTAATCAGTGAG	840	(Essack et al., 2001)
	TEMMR	TTACCAATGCTTAATCAGTGAG		
SHV	SHVMF	TTA ACT CCC TGT TAG CCA	860	(Eckert, Gautier and Arlet, 2006)
	SHVMR	GAT TTG CTG ATT TCG CCC		
CTX-1	CTXMF	GGT TAA AAA ATC ACT GCG TC	1000	(Eckert et al., 2004)
	CTXMR	TTG GTG ACG ATT TTA GCC GC		
